# Role of hepatitis D virus infection in development of hepatocellular carcinoma among chronic hepatitis B patients treated with nucleotide/nucleoside analogues

**DOI:** 10.1038/s41598-021-87679-w

**Published:** 2021-04-14

**Authors:** Tyng-Yuan Jang, Yu-Ju Wei, Ta-Wei Liu, Ming-Lun Yeh, Shu-Fen Liu, Cheng-Ting Hsu, Po-Yao Hsu, Yi-Hung Lin, Po-Cheng Liang, Meng-Hsuan Hsieh, Yu-Min Ko, Yi-Shan Tsai, Kuan-Yu Chen, Ching-Chih Lin, Pei-Chien Tsai, Shu-Chi Wang, Ching-I. Huang, Zu-Yau Lin, Shinn-Cherng Chen, Wan-Long Chuang, Jee-Fu Huang, Chia-Yen Dai, Chung-Feng Huang, Ming-Lung Yu

**Affiliations:** 1 Hepatobiliary Division, Department of Internal Medicine, Kaohsiung Medical University Hospital, Kaohsiung Medical University, 100 Tzyou Road, Kaohsiung, 807 Taiwan; 2 Department of Internal Medicine, Pingtung Hospital, Ministry of Health and Welfare, Ping-Tung, Taiwan; 3 Graduate Institute of Clinical Medicine, College of Medicine, Kaohsiung Medical University, Kaohsiung, Taiwan; 4 Department of Internal Medicine, Kaohsiung Municipal Ta-Tung Hospital, Kaohsiung, Taiwan; 5 Faculty of Internal Medicine and Hepatitis Research Center, School of Medicine, College of Medicine, and Center for Liquid Biopsy and Cohort Research, Kaohsiung Medical University, Kaohsiung, Taiwan; 6 Health Management Center, Kaohsiung Medical University Hospital, Kaohsiung, Taiwan; 7 Department of Occupational and Environmental Medicine, Kaohsiung Medical University Hospital, Kaohsiung, Taiwan; 8 Institute of Biomedical Sciences, National Sun Yat-Sen University, Kaohsiung, Taiwan; 9 Center for Intelligent Drug Systems and Smart Bio-devices (IDS2B) and Department of Biological Science and Technology, College of Biological Science and Technology, National Chiao Tung University, Hsin-Chu, Taiwan

**Keywords:** Hepatitis B, Hepatocellular carcinoma

## Abstract

Hepatitis D virus (HDV) infection increases the risk of hepatocellular carcinoma (HCC) in the natural course of chronic hepatitis B (CHB) patients. Its role in patients treated with nucleotide/nucleoside analogues (NAs) is unclear. We aimed to study the role of hepatitis D in the development of HCC in CHB patients treated with NAs. Altogether, 1349 CHB patients treated with NAs were tested for anti-HDV antibody and RNA. The incidence and risk factors of HCC development were analyzed. Rates of anti-HDV and HDV RNA positivity were 2.3% and 1.0%, respectively. The annual incidence of HCC was 1.4 per 100 person-years after a follow-up period of over 5409.5 person-years. The strongest factor association with HCC development was liver cirrhosis (hazard ratio [HR]/95% confidence interval [CI] 9.98/5.11–19.46, *P* < 0.001), followed by HDV RNA positivity (HR/ CI 5.73/1.35–24.29, *P* = 0.02), age > 50 years old (HR/CI 3.64/2.03–6.54, *P* < 0.001), male gender (HR/CI 2.69/1.29–5.60, *P*: 0.01), and body mass index (BMI, HR/CI 1.11/1.03–1.18, *P* = 0.004). The 5-year cumulative incidence of HCC was 7.3% for patients with HDV RNA negativity compared to that of 22.2% for patients with HDV RNA positivity (*P* = 0.01). In the subgroup of cirrhotic patients, the factors associated with HCC development were HDV RNA positivity (HR/CI 4.45/1.04–19.09, *P* = 0.04) and BMI (HR/CI 1.11/1.03–1.19, *P* = 0.01). HDV viremia played a crucial role in HCC development in CHB patients who underwent NA therapy.

## Introduction

Hepatitis B virus (HBV) infection is one of the major etiological factors of hepatocellular carcinoma (HCC), which imposes a heavy burden on global health. HBV was highly prevalent in adults prior to the launch of the national HBV vaccination programs. It is the leading cause of HCC in Taiwan^1,2^. The etiology of HBV-related HCC is multifactorial and includes virological factors such as HBV DNA levels, HBV genotypes, mutants, and host factors such as age, gender, cirrhosis, and metabolic disorders^3^. Among the risk factors, liver cirrhosis has been the most crucial determinant of HCC. The incidence of HCC increases significantly after the development of liver cirrhosis. The incidence of HCC has been reported to be 0.2 per 100 person-years in inactive carriers, 0.6 per 100 person-years in non-cirrhotic patients, and 3.7 per 100 person-years in patients with compensated cirrhosis^4^. However, antiviral therapy including nucleotide/nucleoside analogues (NAs) is reported to result in an improvement in hepatic inflammation and fibrosis, which in turn reduces the risk of HCC development and mortality^5,6^.

Hepatitis D virus (HDV) is a single-stranded RNA virus and its propagation depends on HBV^7^. The worldwide prevalence of HDV ranges from < 1% to > 50%^8^. Among chronic hepatitis B (CHB) patients, the hepatitis D antibody (anti-HDV) positivity rate is reported to be 2–5% in the general population^9,10^ and up to 68.9% in persons who inject drugs in Taiwan^11^. Chronic hepatitis D (CHD) infection may result in a deterioration of liver function and may also lead to a threefold increase in the risk of HCC in the typical course of the disease^12,13^. Notably, the impact of HDV on HCC occurrence in CHB patients treated with NAs remains unclear. Hence, we aimed to address this issue by enrolling a well-characterized CHB cohort treated with NAs. HDV serology and virology in this cohort were also studied and considered for the evaluation of its impact on HCC development.

## Methods

### Patients

CHB patients allocated to NAs were consecutively enrolled in a medical center in Taiwan from June 2000 to July 2018 in the retrospective study. Patients were followed up from the beginning of NA treatment. The treatment indications for NAs were based on the national health insurance reimbursement regulations of the Ministry of Health and Welfare in Taiwan^2,14^, Briefly, the criteria included (1) HBeAg-negative patients with HBV DNA greater than 2000 IU/mL with persistent alanine aminotransferase abnormality greater than two times the upper limit of normal (ULN) on two occasions 3 months apart, and (2) HBeAg-positive patients with HBV DNA greater than 20,000 IU/mL with ALT abnormality greater than two times the ULN for two occasions 3 months apart, or with ALT > 5 times the ULN, (3) cirrhotic patients with HBV DNA greater than 2000 IU/mL, and (4) liver decompensation, denoted as prothrombin time prolongs > 3 s or a total bilirubin > 2 mg/dL^2^. Patients were excluded if they had any of the following conditions: alcoholism (≥ 20 g daily), coinfection with human immunodeficiency virus or hepatitis C virus (HCV), ongoing interferon-based therapy, pre-existing HCC before the use of NAs, and the use of NAs for chemotherapy prophylaxis. The study was conducted in accordance with the principles of the Declaration of Helsinki of 1975, as revised in 2008. The ethics committee of Kaohsiung Medical University Hospital approved the study. All patients provided informed consent before enrollment.

### Laboratory analyses

Biochemical analyses were performed using a multichannel autoanalyzer (Hitachi Inc., Tokyo, Japan). Hepatitis B surface antigen (HBsAg) was detected using standard quantitative chemiluminescent microparticle immunoassay (ARCHITECT HBsAg, Abbott Diagnostics, Chicago, IL, USA). Serum HBV DNA was examined using a standardized, automated quantitative polymerase chain reaction assay (COBAS TaqMan HBV test, Roche Diagnostics, Branchburg, NJ; detection limit 12 IU/mL)^15^. Anti-HDV antibody was verified at the initiation of treatment with anti-HBV agents. Screening for anti-HDV immunoglobulin G was performed using the anti-HDV enzyme-linked immunosorbent assay kit (General Biologicals Corporation, Hsinchu, Taiwan)^7,10^. HDV RNA was tested in patients with anti-HDV positivity using the LightMix Kit HDV (Roche Life Science, Berlin, Germany) on the Roche LightCycler (Roche Life Science, Berlin, Germany; detection limit: 10 copies/mL)^16^. The fibrosis-4 index was calculated by using the following formula: age (years) × aspartate aminotransferase [U/L]/platelets [10^9^/L] × alanine transaminase [U/L])^1/2^. Liver cirrhosis was diagnosed using transient elastography (FibroScan; Echosens, Paris, France; >12 kPa)^17^, histology, or the presence of laboratory, radiological, endoscopic, or clinical evidence of portal hypertension and/or cirrhosis. HCC was confirmed by clinical or histological diagnosis according to the guidelines of the American Association for the Study of Liver Diseases^18^ and the Asian Pacific Association for the Study of the Liver^19^. Patients visited the clinics every 1 to 3 months depending on the physician’s discretion. Laboratory tests, including aspartate aminotransferase (AST), alanine aminotransferase (ALT), alpha-fetoprotein (AFP), and hepatitis B virus serology/virology were assessed every 3 to 6 months if the patient’s condition was stable. HCC surveillance by abdominal sonography was performed every 3 to 6 months based on the severity of the liver disease.

### Statistical analyses

Frequencies were analyzed between the groups using the chi-square (χ^2^) test with Yates correction or Fisher’s exact test. Data are presented as mean ± standard deviation. Data were compared using analysis of variance, Student’s t-test, or the nonparametric Mann-Whitney U test. A stepwise logistic regression analysis was applied to analyze the characteristics associated with anti-HDV and HDV RNA positivity by analyzing the covariates with *P* values < 0.1 in the univariate analysis. Kaplan–Meier analysis and a log-rank test was performed for the comparison of the cumulative incidence of HCC with respect to various determinants. Cox regression analysis was applied to analyze the factors independently associated with HCC development by analyzing the covariates with *P*-values < 0.2 in the univariate analysis or the factors considered to have potential and clinical relevance. Statistical analyses were performed using IBM SPSS Statistics, version 20 (IBM Corp., Armonk, NY, USA). All statistical analyses were based on two-sided hypothesis tests with statistical significance set at *P* < 0.05.

## Results

### Patient characteristics

**Figure 1:**
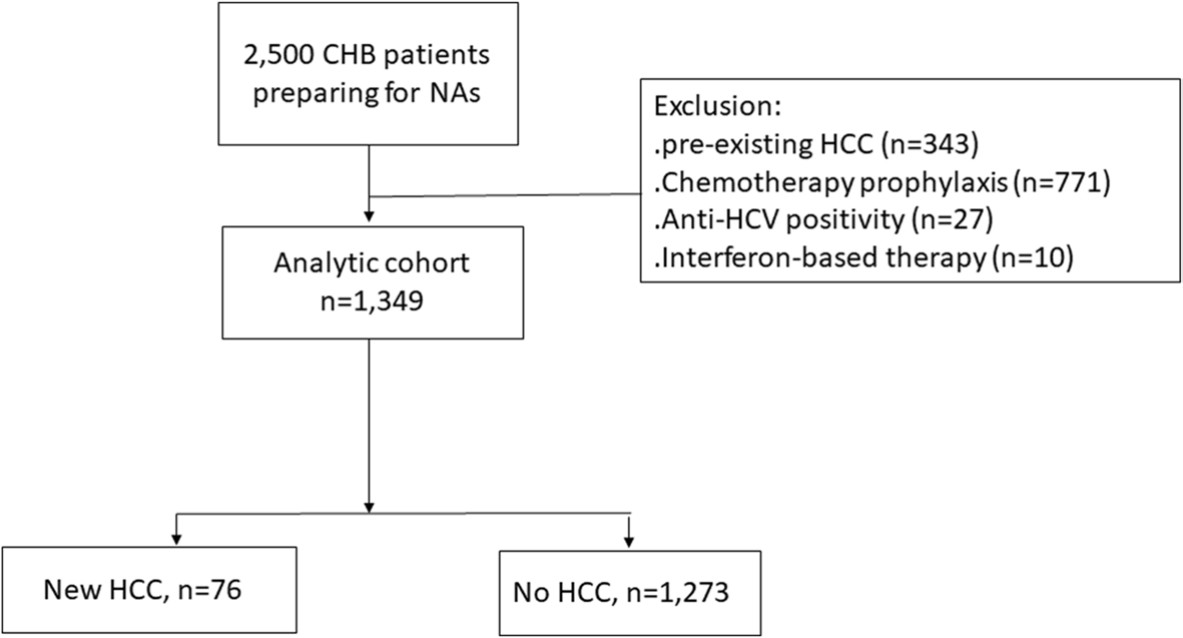
The flowchart of patient enrolment. CHB: chronic hepatitis B; NAs: nucleoside/nucleotide analogue; HCC: hepatocellular carcinoma; ALT: alanine aminotransferase; HCV: hepatitis C.

Altogether, 2500 CHB patients who received NAs were initially recruited. After excluding patients with pre-existing HCC (n = 343), patients using NAs for chemotherapy prophylaxis (n = 771), patients with anti-HCV positivity (n = 27), and patients receiving interferon-based therapy (n = 10), 1349 patients were enrolled for analysis (Fig. 1). The mean age was 48.0 years and 72.4% of the patients were male. The mean HBV DNA level was 5.9 log_10_ IU/mL. Patients with liver cirrhosis accounted for 29.1% (n = 392) of the study population. The rates of anti-HDV and HDV RNA positivity were 2.3% and 1.0%, respectively. The most commonly used NAs were entecavir (45.2%) and tenofovir disoproxil fumarate (12.2%) (Table 1).

**Table 1:** Characteristics of the 1349 chronic hepatitis B patients preparing for NAs treatment.

	All patients (n = 1349)
Age (years, mean (SD))	48.0 (14.1)
Male, n (%)	977 (72.4)
Diabetes, n/N (%)	181/1222 (14.8)
BMI (kg/m^2^, mean [SD])^a^	24.6 (4.1)
AST (IU/L, mean (SD))	299.8 (612.7)
ALT (IU/L, mean (SD))	407.0 (634.9)
Platelet count (×10^3^*u*/L, mean (SD))	166.3 (74.1)
FIB-4 (mean (SD))	5.5 (9.7)
HBV DNA (log_10_ IU/mL, mean (SD))^b^	5.9 (1.9)
HBeAg positivity (%)	523/1339 (39.1)
Anti-HDV positivity, n (%)	31 (2.3)
HDV RNA positivity, n (%)	13 (1.0)
Liver cirrhosis, n (%)	392 (29.1)
ETV/TDF/other NAs^c^, n/n/n	610/164/575

SD: standard deviation; BMI: body mass index; AST: aspartate aminotransferase; ALT: alanine aminotransferase; HBV: hepatitis B virus; HDV: hepatitis D virus; HBeAg: hepatitis B e-antigen; FIB-4: fibrosis-4 index; NAs: nucleoside/nucleotide analogues.

^a^n=1309.

^b^n=1346.

^c^Included lamivudine (n = 401), telbivudine (n = 61), adefovir (n = 34), or NAs combination (n=79).

### Characteristics of patients with HDV infection

Compared to anti-HDV negative patients, those with anti-HDV positivity were older (55.4 years vs. 47.9 years, *P* = 0.003) and had lower HBV DNA levels (4.4 log_10_ IU/mL vs. 6.0 log_10_ IU/mL, *P* = 0.001). Logistic regression analysis revealed that factors associated with anti-HDV positivity included age (odds ratio [OR], 95% confidence interval [CI] 1.04/1.01–1.07; *P*: 0.007) and HBV DNA level (OR/CI 0.71/0.59–0.86; *P* < 0.001) (Supplementary Table 1). There was no difference in terms of the mean HBV DNA level (1.4 log_10_ IU/mL vs. 1.2 log_10_ IU/mL, *P* = 0.35) and the proportion of undetectable HBV DNA (79.5% vs. 90.5%, *P* = 0.28) after 1 year of NA therapy between anti-HDV negative and positive patients.

Compared to HDV RNA negative patients, those with HDV RNA positivity had lower baseline HBV DNA levels (4.0 log_10_ IU/mL vs. 5.9 log_10_ IU/mL, *P* = 0.003). Logistic regression analysis revealed that the only factor independently associated with HDV RNA positivity was a baseline HBV DNA level (OR/CI 0.63/0.48–0.83; *P* < 0.001) (Supplementary Table 2).

### Cumulative incidence and risk factors of HCC development

**Figure 2:**
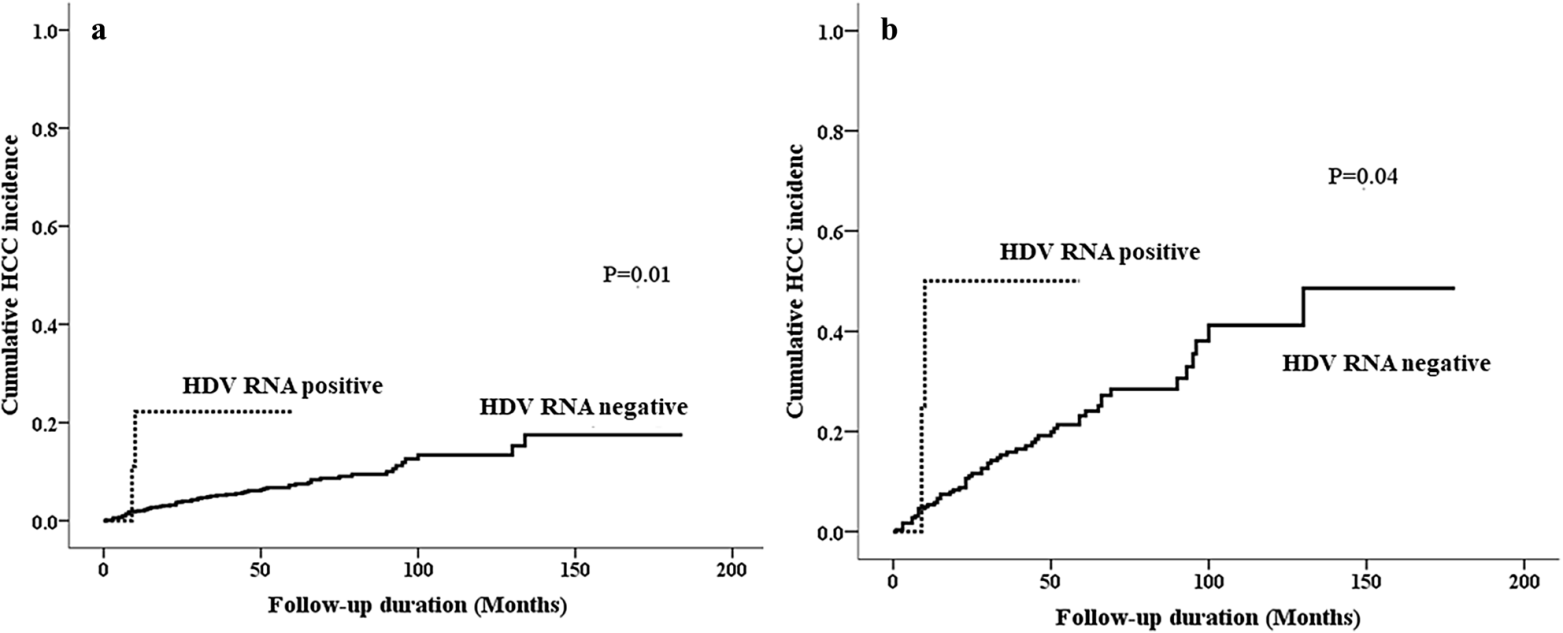
(**a**) Cumulative incidence of HCC in CHB patients with HDV RNA (+) and HDV RNA (−). (**b**) Cumulative incidence of HCC in cirrhotic patients with HDV RNA (+) and HDV RNA (−).

Seventy-six (5.6%) patients developed HCC over a follow-up period of 5409.5 person-years (range: 1.0–15.0 years; annual incidence: 1.4%). The cumulative incidence of HCC was 2.3%, 5.4%, and 7.5% at the 1-year, 3-year, and 5-year follow-ups, respectively. Compared to patients without HCC development, patients with HCC had higher proportions of: age over 50 years (69.7% vs. 40.9%, *P* < 0.001), male gender (85.5% vs. 71.6%, *P* = 0.008), liver cirrhosis (81.6% vs. 25.9%, *P* < 0.001), higher body mass index (BMI) (26.1 kg/m^2^ vs. 24.5 kg/m^2^, *P* = 0.001), lower platelet count (117.1×10^3^ *u*/L vs. 169.3×10^3^ *u*/L, P < 0.001), and a substantially higher proportion of HDV RNA positivity (2.6% vs. 0.9%, *P* = 0.16, Table 2). Cox-regression analysis revealed that the strongest factor associated with HCC development was liver cirrhosis (hazard ratio [HR]: 95% confidence interval [CI] 9.98/5.11–19.46, *P* < 0.001), followed by HDV RNA positivity (HR: 5.73, CI 1.35–24.29, *P* = 0.02), age over 50 years (HR: 3.64, CI 2.03–6.54, *P* < 0.001), male gender (HR: 2.69, CI 1.29–5.60, *P*=0.01), and BMI (HR: 1.11, CI 1.03–1.18, *P* = 0.004, Table 2). The 1-year, 3-year, and 5-year cumulative incidence rates of HCC were 2.1%, 5.3%, and 7.3%, respectively, in anti-HDV (−) or anti-HDV (+)/HDV RNA (−) patients when compared with the incidence rates of 22.2%, 22.2%, and 22.2%, respectively, in HDV RNA (+) patients (log-rank *P* = 0.01, Fig. 2a). Among the 31 anti-HDV-positive patients, there was no identifiable factor associated with HCC development (Supplementary Table 3).

**Table 2:** Factors associated with the new onset of HCC after HBV NAs use.

New onset HCC	Yes, n = 76	No, n = 1273	P value	Cox-regression analysis		
				HR	95% C.I.	P value
Age > 50 years old, n (%)	53 (69.7)	517 (40.6)	< 0.001	3.64	2.03–6.54	< 0.001
Male, n (%)	65 (85.5)	912 (71.6)	0.008	2.69	1.29–5.60	0.01
Diabetes, n/N (%)	17/68 (25.0)	164/1154 (14.2)	0.02	1.20	0.64–2.25	0.58
BMI (kg/m^2^, mean [SD])^a^	26.1 (4.1)	24.5 (4.1)	0.001	1.10	1.03–1.18	0.004
Platelet count (×10^3^*u*/L, mean (SD))	117.1 (70.2)	169.3 (73.3)	< 0.001	0.997	0.995–1.000	0.07
AST (IU/L, mean (SD))	146.2 (183.9)	309.1 (628.3)	< 0.001	1.001	1.000–1.003	0.14
ALT (IU/L, mean (SD))	149.7 (186.0)	422.6 (649.1)	< 0.001	0.997	0.995–1.000	0.03
Creatinine (mg/dL, mean (SD))	1.0 (0.8)	1.0 (1.1)	0.81			
HBV DNA (log10 IU/mL, mean (SD))^b^	5.6 (1.7)	5.9 (1.9)	0.12	0.96	0.81–1.14	0.61
HBV DNA > 2000 IU/mL, n/N (%)	65/75 (86.7)	1134/1271 (89.2)	0.45			
HDV RNA positivity, n (%)	2 (2.6)	11 (0.9)	0.16	5.73	1.35–24.29	0.02
HBeAg positivity, n/N (%)	22/75 (29.3)	501/1264 (39.6)	0.09	1.21	0.60–2.45	0.60
Liver cirrhosis, n (%)	62 (81.6)	230 (25.9)	< 0.001	9.98	5.11–19.46	< 0.001
ETV/TDF/other NAs, n/n/n	41/5/30	569/159/545	0.17	1.04	0.67–1.61	0.88
Duration of NAs usage (months, mean (SD))	38.4 (24.0)	26.0 (23.4)	–			
Follow-up period (months, mean (SD))	36.7 (35.1)	48.8 (37.6)	–			
Follow-up period (months, median (range))	23.5 (3–130)	41.0 (3–184)	–			

SD: standard deviation; BMI: body mass index; AST: aspartate aminotransferase; ALT: alanine aminotransferase; HBsAg: hepatitis B surface antigen; HBV: hepatitis B virus; HDV: hepatitis D virus; HBeAg: hepatitis B e-antigen; FIB-4: fibrosis-4 index; HCC: hepatocellular carcinoma; NAs: nucleotide analogues; ETV: entecavir; TDF: tenofovir disoproxil fumarate; HR: hazard ratio; CI: confidence interval.

^a^n = 1309.

^b^n = 1346.

### Risk factors of HCC development in patients with or without liver cirrhosis

Since underlying liver cirrhosis was the most important predictive factor of HCC, we analyzed the occurrence of HCC stratified by liver cirrhosis. Sixty-two out of 392 cirrhotic patients (15.8%) developed HCC over a follow-up period of 1355.7 person-years (annual incidence: 4.6%). The cumulative incidence rates of HCC were 6.0%, 16.4%, and 23.5% at the 1-year, 3-year, and 5-year follow-ups, respectively. Compared to patients without HCC development, patients with HCC had substantially higher BMI (26.1 kg/m^2^ vs. 25.2 kg/m^2^, *P* = 0.13) and a substantially higher proportion of HDV RNA positivity (3.2% vs. 0.9%, *P* = 0.18), although the differences did not reach the conventional level of statistical significance (Table 3). Cox regression analysis revealed that the factors associated with HCC development were BMI (HR/CI 1.11/1.03–1.19, *P* = 0.01) and HDV RNA positivity (HR/CI 4.45/1.04–19.09, *P* = 0.04, Table 3). In cirrhotic patients, the 1-year, 3-year, and 5-year cumulative incidence rates of HCC were 5.4%, 15.9%, and 23.1%, respectively, in anti-HDV (−) or anti-HDV (+)/HDV RNA (−) patients when compared with the incidence rates of 50.0%, 50.0%, and 50.0%, respectively, in anti-HDV (+)/HDV RNA (+) patients (log-rank *P* = 0.04, Fig. 2b).

**Table 3:** Factors associated with the new onset of HCC after HBV NAs use in cirrhotic patients.

	With HCC (n = 62)	Without HCC (n = 330)	P value	Cox-regression analysis		
				HR	95% C.I	P value
Age > 50 years old, n (%)	41 (66.1)	192 (58.2)	0.24			
Male, n (%)	53 (85.5)	242 (73.4)	0.05	1.99	0.88–4.48	0.10
Diabetes, n/N (%)	12/54 (22.4)	63/303 (20.8)	0.86			
BMI (kg/m^2^, mean [SD])^a^	26.1 (3.8)	25.2 (4.1)	0.13	1.11	1.03–1.19	0.01
Platelet count (×10^3^*u*/L, mean (SD))	111.0 (71.3)	123.3 (58.1)	0.15	1.00	0.99–1.00	0.09
AST (IU/L, mean (SD))	154.9 (201.4)	174.3 (308.8)	0.64			
ALT (IU/L, mean (SD))	152.1 (201.5)	182.5 (305.9)	0.45			
Creatinine (mg/dL, mean (SD))	1.0 (0.9)	1.1 (1.2)	0.65			
HBV DNA (log10 IU/mL, mean (SD))	5.6 (1.7)	5.4 (1.8)	0.38			
HBV DNA > 2000 IU/mL, n/N (%)	54 (87.1)	28 (86.4)	1.00			
HDV RNA positivity, n (%)	2 (3.2)	3 (0.9)	0.18	4.45	1.04–19.09	0.04
HBeAg positivity, n/N (%)	17/61 (27.9)	65/328 (19.8)	0.17	0.97	0.47–2.01	0.94

SD: standard deviation; BMI: body mass index; AST: aspartate aminotransferase; ALT: alanine aminotransferase; HBsAg: hepatitis B surface antigen; HBV: hepatitis B virus; HDV: hepatitis D virus; HBeAg: hepatitis B e-antigen; FIB-4: fibrosis-4 index; HCC: hepatocellular carcinoma; NAs: nucleotide analogues; HR: hazard ratio; CI: confidence interval.

^a^n = 384.

Fourteen out of 957 non-cirrhotic patients (1.5%) developed HCC over a follow-up period of 4051.3 person-years (annual incidence: 0.35%). The cumulative incidence rates of HCC in non-cirrhotic patients were 0.8%, 1.2%, and 1.2%, respectively, at the 1-year, 3-year, and 5-year follow-ups, respectively. Compared to patients without HCC development, those with HCC represented a higher proportion of patients over 50 years (85.7% vs. 34.5%, *P* < 0.001), diabetes (35.7% vs. 11.9%, *P* = 0.02) and lower platelet counts (145.5×10^3^ vs. 185.7×10^3^ *u*/L, *P* = 0.05). Cox regression analysis revealed that the factors associated with HCC development were an age over 50 years (HR/CI 13.26/1.60–109.75, *P* < 0.001) and platelet count (HR/CI 0.99/0.98–1.00, *P* = 0.04) (Supplementary Table 4).

## Discussion

In the present study, we observed that the annual incidence of HCC was 1.4% in CHB patients treated with NAs. As expected, liver cirrhosis was the most important risk factor associated with HCC occurrence. We demonstrated that coinfection with HDV also played an essential role in HCC development, although the prevalence was low in Taiwanese patients. Compared to patients without concurrent HDV infection, those with HDV viremia had a nearly sixfold risk of HCC development.

HBV infection induces hepatic inflammation, which leads to liver cirrhosis and HCC. The HBV DNA level has a dose-dependent association with HCC development in the typical course of the disease^20^. The other risk factors of HBV-related HCC include liver cirrhosis, age, gender, and metabolic factors^21,22^. Several models have assessed the risk of HCC in CHB patients without antiviral therapy^23,24^. Moreover, emerging evidence has shown that HBV DNA suppression with NAs^25,26^ or interferon^27,28^ significantly reduced the HCC risk when compared with untreated controls. Currently, NAs are the mainstream agents for CHB therapy. They have reduced the HCC risk by 34–78% according to various studies^29^. However, HCC continues to develop under viral suppression. Some HCC prediction models have also been created in patients undergoing treatment with NAs^30,31^ and the pre-treatment viral loads were not found to be as crucial as those in NA-naïve patients. Underlying liver cirrhosis was reported to be the most critical determinant of HCC^32^, which is consistent with the results of the present study. The reported annual incidence of HCC was 0.3–1.2% in non-cirrhotic CHB patients and 1.8–6.0% in cirrhotic CHB patients undergoing NA therapy^33^. We observed a similar incidence of HCC in our patient cohort, with an annual incidence of 0.4% in non-cirrhotic patients and 4.6% in cirrhotic patients. Continuous surveillance of HCC remains mandatory in CHB patients receiving antiviral therapy, and it is crucial in cirrhotic patients.

The prevalence of HDV in the general population is low. It has been decreasing in Taiwan in recent decades^9,10^. The seroprevalence of anti-HDV and HDV RNA was 2.3% and 1%, respectively, in the present study, which is similar to that observed in a recent report in Taiwan^9^. HDV would increase the risk of HCC in the typical course of the disease^13,22^. However, debates continue regarding this issue^9,34^. There is no potent treatment against HDV infection. To date, interferon has been the standard of care^35^ and NAs are not effective in treating CHD^36^. However, NAs remain the mainstay for managing CHB. The impact of HDV on HCC has rarely been elucidated in NA-treated cohorts. Brancaccio et al. conducted a case-control study that disclosed that despite long-term NAs use, an increased risk of HCC remained in HDV-infected patients compared to those with HBV mono-infection^37^. In the current long-term observational study with a larger Asian cohort, we consistently demonstrated that HDV viremia increases the risk of HCC. This finding was also in line with the results from another recent study^38^. The aforementioned study by Kamal et al*.* suggested that HDV viremia might increase the incidence of liver-related events such as HCC, hepatic decompensation, and death. Notably, the majority of the patients were treated with interferon, while only a few patients received NAs^38^. In the present NA-based cohort study, we observed that one-fifth of the patients with HDV viremia had developed HCC at the 5-year follow-up. Moreover, 50% of the HDV-RNA positive cirrhotic patients eventually developed HCC. Currently, there is no potent antiviral agent to treat HDV infection. Given the high risk of HCC development, we suggest that each HDV-infected subject should be monitored regularly and closely until effective or curative anti-HDV agents can be prescribed in the clinical setting.

The present study has some limitations. Due to the low prevalence of HDV infection in the region where the study was conducted, the retrospective cohort was limited by relatively few index patients with HDV infection, particularly in non-cirrhotic subjects. This may have restricted the cumulative clinical impact of HDV on HCC. As with the majority of other studies, the current study aimed to identify baseline factors including HDV status rather than the sequential parameters predictive of HCC. We failed to provide information regarding cirrhotic development or resolution at the time of event occurrence or patient censoring. In addition, the follow-up duration was more condensed in HDV-infected patients. Although the follow-up duration was condensed in the HDV group, the HCC incidence was significantly higher than that in HBV mono-infected patients during the relatively brief observation period. The difference in HCC incidence between the groups was postulated to be more pronounced if the follow-up duration was prolonged in the HDV group.

In conclusion, the results of the present study suggest that HDV viremia plays an essential role in HCC development in CHB patients treated with NAs. HDV testing should be proposed for each HBV subject even when HBV replication is under control by NAs. We hope that these results will promote further validated studies, particularly in regions where HDV infection is widespread.

## Supplementary Information

Supplementary Information.

## References

[1] Yang JF, Lin CI, Huang JF, Dai CY, Lin WY, Ho CK (2010). Viral hepatitis infections in southern Taiwan: A multicenter community-based study. Kaohsiung J. Med. Sci..

[2] Chien RN, Kao JH, Peng CY, Chen CH, Liu CJ, Huang YH (2019). Taiwan consensus statement on the management of chronic hepatitis B. J. Formos Med. Assoc..

[3] El-Serag HB (2012). Epidemiology of viral hepatitis and hepatocellular carcinoma. Gastroenterology.

[4] Fattovich G, Bortolotti F, Donato F (2008). Natural history of chronic hepatitis B: Special emphasis on disease progression and prognostic factors. J. Hepatol..

[5] Grossi G, Vigano M, Loglio A, Lampertico P (2017). Hepatitis B virus long-term impact of antiviral therapy nucleot(s)ide analogues (NUCs). Liver Int..

[6] Chen VL, Yeh ML, Le AK, Jun M, Saeed WK, Yang JD (2018). Anti-viral therapy is associated with improved survival but is underutilised in patients with hepatitis B virus-related hepatocellular carcinoma: Real-world east and west experience. Aliment Pharmacol. Ther..

[7] Mentha N, Clement S, Negro F, Alfaiate D (2019). A review on hepatitis D: From virology to new therapies. J. Adv. Res..

[8] Vlachogiannakos J, Papatheodoridis GV (2020). New epidemiology of hepatitis delta. Liver Int..

[9] Lin HH, Lee SS, Yu ML, Chang TT, Su CW, Hu BS (2015). Changing hepatitis D virus epidemiology in a hepatitis B virus endemic area with a national vaccination program. Hepatology.

[10] Jang TY, Wei YJ, Hsu CT, Hsu PY, Liu TW, Lin YH (2020). Serial serologic changes of hepatitis D virus in chronic hepatitis B patients receiving nucleos(t)ides analogues therapy. J. Gastroenterol. Hepatol..

[11] Hsieh MH, Wang SC, Hsieh MY, Huang CF, Yeh ML, Yang JF (2016). Hepatitis D virus infections among injecting drug users with and without human immunodeficiency virus infection in Taiwan. Kaohsiung J. Med. Sci..

[12] Tamura I, Kurimura O, Koda T, Ichimura H, Katayama S, Kurimura T (1993). Risk of liver cirrhosis and hepatocellular carcinoma in subjects with hepatitis B and delta virus infection: A study from Kure, Japan. J. Gastroenterol. Hepatol..

[13] Alfaiate D, Clement S, Gomes D, Goossens N, Negro F (2020). Chronic hepatitis D and hepatocellular carcinoma: A systematic review and meta-analysis of observational studies. J. Hepatol..

[14] https://www.nhi.gov.tw/BBS_Detail.aspx?n=73CEDFC921268679&sms=D6D5367550F18590&s=66360DBE1F9DFA41. Accessed on 2019.09.07.

[15] Lin YY, Huang JF, Liu SF, Yu ML, Tsai CH, Yang JF (2009). Performance characteristics of two real-time PCR assays for quantification of hepatitis B virus DNA. Scand. J. Infect. Dis..

[16] Ghamari S, Alavian SM, Rizzetto M, Olivero A, Smedile A, Khedive A (2013). Prevalence of hepatitis delta virus (HDV) infection in chronic hepatitis B patients with unusual clinical pictures. Hepat. Mon..

[17] Castera L, Vergniol J, Foucher J, Le Bail B, Chanteloup E, Haaser M (2005). Prospective comparison of transient elastography, Fibrotest, APRI, and liver biopsy for the assessment of fibrosis in chronic hepatitis C. Gastroenterology.

[18] Heimbach JK, Kulik LM, Finn RS, Sirlin CB, Abecassis MM, Roberts LR (2018). AASLD guidelines for the treatment of hepatocellular carcinoma. Hepatology.

[19] Omata M, Cheng AL, Kokudo N, Kudo M, Lee JM, Jia J (2017). Asia-Pacific clinical practice guidelines on the management of hepatocellular carcinoma: A 2017 update. Hepatol. Int..

[20] Chen CJ, Yang HI (2011). Natural history of chronic hepatitis B REVEALed. J. Gastroenterol. Hepatol..

[21] Kasmari AJ, Welch A, Liu G, Leslie D, McGarrity T, Riley T (2017). Independent of cirrhosis, hepatocellular carcinoma risk is increased with diabetes and metabolic syndrome. Am. J. Med..

[22] Romeo R, Del Ninno E, Rumi M, Russo A, Sangiovanni A, de Franchis R (2009). A 28-year study of the course of hepatitis Delta infection: A risk factor for cirrhosis and hepatocellular carcinoma. Gastroenterology.

[23] Yang HI, Yuen MF, Chan HL, Han KH, Chen PJ, Kim DY (2011). Risk estimation for hepatocellular carcinoma in chronic hepatitis B (REACH-B): Development and validation of a predictive score. Lancet Oncol..

[24] Yang HI, Yeh ML, Wong GL, Peng CY, Chen CH, Trinh HN (2020). Real-world effectiveness from the Asia Pacific Rim liver consortium for HBV risk score for the prediction of hepatocellular carcinoma in chronic hepatitis B patients treated with oral antiviral therapy. J. Infect. Dis..

[25] Lin CL, Kao JH (2015). Perspectives and control of hepatitis B virus infection in Taiwan. J. Formos Med. Assoc..

[26] Wu CY, Lin JT, Ho HJ, Su CW, Lee TY, Wang SY (2014). Association of nucleos(t)ide analogue therapy with reduced risk of hepatocellular carcinoma in patients with chronic hepatitis B: A nationwide cohort study. Gastroenterology.

[27] Lin SM, Yu ML, Lee CM, Chien RN, Sheen IS, Chu CM (2007). Interferon therapy in HBeAg positive chronic hepatitis reduces progression to cirrhosis and hepatocellular carcinoma. J. Hepatol..

[28] Yeh ML, Huang JF, Dai CY, Yu ML, Chuang WL (2019). Pharmacokinetics and pharmacodynamics of pegylated interferon for the treatment of hepatitis B. Expert Opin. Drug Metab. Toxicol..

[29] Lin CL, Kao JH (2018). Review article: The prevention of hepatitis B-related hepatocellular carcinoma. Aliment Pharmacol. Ther..

[30] Hsu YC, Yip TC, Ho HJ, Wong VW, Huang YT, El-Serag HB (2018). Development of a scoring system to predict hepatocellular carcinoma in Asians on antivirals for chronic hepatitis B. J. Hepatol..

[31] Kim JH, Kim YD, Lee M, Jun BG, Kim TS, Suk KT (2018). Modified PAGE-B score predicts the risk of hepatocellular carcinoma in Asians with chronic hepatitis B on antiviral therapy. J. Hepatol..

[32] Liu L (2020). Clinical features of hepatocellular carcinoma with hepatitis B virus among patients on Nucleos(t) ide analog therapy. Infect. Agent Cancer.

[33] Hiramatsu N, Yamada R, Takehara T (2016). The suppressive effect of nucleos(t)ide analogue treatment on the incidence of hepatocellular carcinoma in chronic hepatitis B patients. J. Gastroenterol. Hepatol..

[34] Huo TI, Wu JC, Lai CR, Lu CL, Sheng WY, Lee SD (1996). Comparison of clinico-pathological features in hepatitis B virus-associated hepatocellular carcinoma with or without hepatitis D virus superinfection. J. Hepatol..

[35] Krause A, Haberkorn U, Mier W (2018). Strategies for the treatment of HBV/HDV. Eur. J. Pharmacol..

[36] Petersen J, Thompson AJ, Levrero M (2016). Aiming for cure in HBV and HDV infection. J. Hepatol..

[37] Brancaccio G, Fasano M, Grossi A, Santantonio TA, Gaeta GB (2019). Clinical outcomes in patients with hepatitis D, cirrhosis and persistent hepatitis B virus replication, and receiving long-term tenofovir or entecavir. Aliment Pharmacol. Ther..

[38] Kamal H, Westman G, Falconer K, Duberg AS, Weiland O, Haverinen S (2020). Long-term study of hepatitis D infection at secondary care centers: The impact of viremia on liver-related outcomes. Hepatology.

