# Supplementary material for: Role of hepatitis D virus infection in development of hepatocellular carcinoma among chronic hepatitis B patients treated with nucleotide/nucleoside analogues
Source: Sci Rep. 2021 Apr 14;11:8184. doi: 10.1038/s41598-021-87679-w (PMC8047028; doi:10.1038/s41598-021-87679-w)
Supplement: Supplementary file 1 — Supplementary Information. [file 41598_2021_87679_MOESM1_ESM.docx]

Supplementary table 1. Characteristics of patients with and without anti-HDV positivity

| **Anti-HDV positivity** | **Yes, n=31** | **No, n=1,318** | ***P* value** | **Logistic regression analysis** | | |
| --- | --- | --- | --- | --- | --- | --- |
|  |  |  |  | **OR** | **95% C.I.** | ***P* value** |
| Age (years, mean (SD)) | 55.4 (14.0) | 47.9 (14.0) | 0.003 | 1.04 | 1.01-1.07 | 0.007 |
| Male, n (%) | 20 (64.5) | 957 (72.6) | 0.31 |  |  |  |
| Diabetes, n/N (%) | 7/30 (23.3) | 174/1192 (14.6) | 0.19 |  |  |  |
| BMI (kg/m^2^, mean [SD])^†^ | 24.9 (4.4) | 24.6 (4.1) | 0.73 |  |  |  |
| AST (IU/L, mean (SD)) | 316.9 (455.4) | 299.4 (616.1) | 0.88 |  |  |  |
| ALT (IU/L, mean (SD)) | 396.9 (524.5) | 407.2 (637.5) | 0.93 |  |  |  |
| Platelet count (x10^3^*u*/L, mean (SD)) | 157.8 (86.9) | 166.5 (73.8) | 0.53 |  |  |  |
| Creatinine (mg/dL, mean (SD)) | 1.4 (2.0) | 1.0 (1.1) | 0.39 |  |  |  |
| HBV DNA level^‡^ (log_10_ IU/mL, mean (SD)) | 4.4 (2.3) | 6.0 (1.9) | 0.001 | 0.71 | 0.59-0.86 | <0.001 |
| Detectable HBV DNA at year 1, n/N (%) | 2/21 (9.5) | 210/1023 (20.5) | 0.28 |  |  |  |
| Liver cirrhosis, n (%) | 12 (38.7) | 380 (28.8) | 0.23 |  |  |  |

Note: SD, standard deviation; BMI, body mass index; AST, aspartate aminotransferase; ALT, alanine aminotransferase; HBsAg, hepatitis B surface antigen; HBV, hepatitis B virus; HDV, hepatitis D virus; OR, odds ratio; CI, confidence interval. ^†^n=1309. ^‡^n=1346.

Supplementary table 2. Characteristics of patients with and without HDV RNA positivity

| **HDV RNA positivity** | **Yes, n=13** | **No, n=1,336**^§^ | ***P* value** | **Logistic regression analysis** | | |
| --- | --- | --- | --- | --- | --- | --- |
|  |  |  |  | **OR** | **95% C.I.** | ***P* value** |
| Age (years, mean (SD)) | 50.7 (14.5) | 48.0 (14.1) | 0.45 |  |  |  |
| Male, n (%) | 9 (69.2) | 968 (72.5) | 0.76 |  |  |  |
| Diabetes, n/N (%) | 1/13 (7.7) | 180/1209 (14.9) | 0.71 |  |  |  |
| BMI (kg/m^2^, mean [SD])^†^ | 25.5 (3.8) | 24.6 (4.1) | 0.40 |  |  |  |
| AST (IU/L, mean (SD)) | 304.4 (359.1) | 299.7 (614.8) | 0.92 |  |  |  |
| ALT (IU/L, mean (SD)) | 355.2 (433.8) | 407.5 (636.7) | 0.73 |  |  |  |
| Platelet count (x10^3^*u*/L, mean (SD)) | 184.1 (111.6) | 166.1 (73.7) | 0.69 |  |  |  |
| Creatinine (mg/dL, mean (SD)) | 2.0 (3.0) | 1.0 (1.1) | 0.33 |  |  |  |
| HBV DNA level^‡^ (log_10_ IU/mL, mean (SD)) | 4.0 (2.2) | 5.9 (1.9) | 0.003 | 0.63 | 0.48-0.83 | <0.001 |
| Liver cirrhosis, n (%) | 5 (38.5) | 387 (29.0) | 0.54 |  |  |  |

Note: SD, standard deviation; BMI, body mass index; AST, aspartate aminotransferase; ALT, alanine aminotransferase; HBsAg, hepatitis B surface antigen; HBV, hepatitis B virus; HDV, hepatitis D virus; OR, odds ratio; CI, confidence interval. ^†^n=1309. ^‡^n=1346. ^§^The group includes both anti-HDV negative patients (n=1318) and anti-HDV positive/ HDV RNA negative patients (n=18)

Supplementary table 3. Factors associated with HCC development among anti-HDV positive patients

|  | **HCC (+) (n=5)** | **HCC (-) (n=26)** | ***P* value** | **Cox regression analysis** | | |
| --- | --- | --- | --- | --- | --- | --- |
|  |  |  |  | **HR** | **95% C.I.** | ***P* value** |
| Age (years, mean (SD)) | 57.0 (12.9) | 55.1 (14.5) | 0.77 |  |  |  |
| Male, n (%) | 4 (80.0) | 16 (61.5) | 0.63 |  |  |  |
| Diabetes, n/N (%) | 2 (40.0) | 5 (20.0) | 0.57 |  |  |  |
| BMI (kg/m^2^, mean [SD]) | 26.9 (7.3) | 24.4 (3.7) | 0.60 |  |  |  |
| AST (IU/L, mean (SD)) | 96.6 (43.9) | 359.3 (486.9) | 0.83 |  |  |  |
| ALT (IU/L, mean (SD)) | 109.0 (91.5) | 452.2 (555.8) | 0.56 |  |  |  |
| Platelet count (x10^3^*u*/L, mean (SD)) | 85.4 (32.8) | 172.2 (87.4) | 0.01 | 0.97 | 0.94-1.01 | 0.10 |
| Creatinine (mg/dL, mean (SD)) | 2.4 (3.3) | 1.2 (1.7) | 0.55 |  |  |  |
| HBV DNA level (log_10_ IU/mL, mean (SD)) | 4.4 (1.9) | 4.4 (2.4) | 0.95 |  |  |  |
| HBeAg positivity, n (%) | 1 (20.0) | 0 (0) | 0.16 |  |  |  |
| Liver cirrhosis, n (%) | 3 (60.0) | 9 (34.6) | 0.35 |  |  |  |

Note: SD = standard deviation; BMI = body mass index; AST = aspartate aminotransferase; ALT = alanine aminotransferase; HBsAg = hepatitis B surface antigen; HBV = hepatitis B virus; HDV = hepatitis D virus; HBeAg: hepatitis B e-antigen; HR: hazard ratio; CI: confidence interval

|  | **With HCC (n=14)** | **Without HCC (n=943)** | **P value** | **Cox-regression analysis** | | |
| --- | --- | --- | --- | --- | --- | --- |
|  |  |  |  | **HR** | **95% C.I.** | **P value** |
| Age > 50 years old, n (%) | 12 (85.7) | 325 (34.5) | <0.001 | 13.26 | 1.60-109.75 | 0.002 |
| Male, n (%) | 12 (85.7) | 670 (71.0) | 0.37 |  |  |  |
| Diabetes, n/N (%) | 5/14 (35.7) | 101/851 (11.9) | 0.02 | 2.35 | 0.67-8.27 | 0.18 |
| BMI (kg/m^2^, mean [SD])^†^ | 25.9 (5.5) | 24.3 (4.0) | 0.33 |  |  |  |
| Platelet count (x10^3^*u*/L, mean (SD)) | 145.5 (59.8) | 185.7 (71.2) | 0.05 | 0.99 | 0.98-1.00 | 0.04 |
| AST (IU/L, mean (SD)) | 107.5 (52.7) | 356.4 (700.9) | 0.20 |  |  |  |
| ALT (IU/L, mean (SD)) | 139.5 (94.0) | 507.0 (713.6) | 0.02 | 1.00 | 0.99-1.00 | 0.10 |
| Creatinine (mg/dL, mean (SD)) | 0.9 (0.2) | 1.0 (1.1) | 0.43 |  |  |  |
| HBV DNA^‡^(log10 IU/mL, mean (SD)) | 5.7 (2.3) | 6.1 (1.9) | 0.44 |  |  |  |
| HBV DNA > 2000 IU/mL, n/N (%) | 11/13 (84.6) | 849/941 (90.2) | 0.37 |  |  |  |
| HDV RNA positivity, n (%) | 0 (0) | 8 (0.8) | 1.00 |  |  |  |
| HBeAg positivity, n/N (%) | 5/14 (35.7) | 436/936 (46.6) | 0.59 |  |  |  |

Supplementary table 4. Factors associated with the new onset of HCC after HBV NAs use in non-cirrhotic patients

Note: SD = standard deviation; BMI = body mass index; AST = aspartate aminotransferase; ALT = alanine aminotransferase; HBsAg = hepatitis B surface antigen; HBV = hepatitis B virus; HDV = hepatitis D virus; HBeAg: hepatitis B e-antigen; FIB-4: fibrosis-4 index; HCC: hepatocellular carcinoma; NAs: nucleotide analogues; HR: hazard ratio; CI: confidence interval, ^†^n=925, ^‡^n=954
